# The REporting of Studies Conducted Using Observational Routinely-Collected Health Data (RECORD) Statement: Methods for Arriving at Consensus and Developing Reporting Guidelines

**DOI:** 10.1371/journal.pone.0125620

**Published:** 2015-05-12

**Authors:** Stuart G. Nicholls, Pauline Quach, Erik von Elm, Astrid Guttmann, David Moher, Irene Petersen, Henrik T. Sørensen, Liam Smeeth, Sinéad M. Langan, Eric I. Benchimol

**Affiliations:** 1 Department of Epidemiology and Community Medicine, University of Ottawa, Ottawa, Canada; 2 Children′s Hospital of Eastern Ontario Research Institute, Ottawa, Canada; 3 Institute for Clinical Evaluative Sciences, Toronto, Canada; 4 Cochrane Switzerland, Institute of Social and Preventive Medicine, University of Lausanne, Lausanne, Switzerland; 5 Hospital for Sick Children, Department of Paediatrics and Institute of Health Policy, Management and Evaluation, University of Toronto, Toronto, Canada; 6 Ottawa Hospital Research Institute, Ottawa, Canada; 7 Department of Primary Care and Population Health, University College London, London, United Kingdom; 8 Department of Clinical Epidemiology, Aarhus University, Aarhus, Denmark; 9 London School of Hygiene and Tropical Medicine, London, United Kingdom; 10 Department of Pediatrics, Children′s Hospital of Eastern Ontario, University of Ottawa, Ottawa, Canada; National Taiwan University, TAIWAN

## Abstract

**Objective:**

Routinely collected health data, collected for administrative and clinical purposes, without specific *a priori* research questions, are increasingly used for observational, comparative effectiveness, health services research, and clinical trials. The rapid evolution and availability of routinely collected data for research has brought to light specific issues not addressed by existing reporting guidelines. The aim of the present project was to determine the priorities of stakeholders in order to guide the development of the **RE**porting of studies **C**onducted using **O**bservational **R**outinely-collected health **D**ata (**RECORD**) statement.

**Methods:**

Two modified electronic Delphi surveys were sent to stakeholders. The first determined themes deemed important to include in the RECORD statement, and was analyzed using qualitative methods. The second determined quantitative prioritization of the themes based on categorization of manuscript headings. The surveys were followed by a meeting of RECORD working committee, and re-engagement with stakeholders via an online commentary period.

**Results:**

The qualitative survey (76 responses of 123 surveys sent) generated 10 overarching themes and 13 themes derived from existing STROBE categories. Highest-rated overall items for inclusion were: Disease/exposure identification algorithms; Characteristics of the population included in databases; and Characteristics of the data. In the quantitative survey (71 responses of 135 sent), the importance assigned to each of the compiled themes varied depending on the manuscript section to which they were assigned. Following the working committee meeting, online ranking by stakeholders provided feedback and resulted in revision of the final checklist.

**Conclusions:**

The RECORD statement incorporated the suggestions provided by a large, diverse group of stakeholders to create a reporting checklist specific to observational research using routinely collected health data. Our findings point to unique aspects of studies conducted with routinely collected health data and the perceived need for better reporting of methodological issues.

## Introduction

The entry of health care into the electronic age has led to clinical benefits[1–3], as well as the proliferation of large data repositories containing routinely collected health data. These are defined as data collected for administrative and clinical purposes, without specific *a priori* research questions[3, 4]. Examples of such routine data collection include, but are not limited to, health claims data, primary care and hospital electronic health records, and disease registries such as those established for audit purposes.

While these data are collected primarily for healthcare administration or clinical management, the nature and scale of the data make them potentially exciting resources for research. Routinely collected data now are being used for observational, comparative effectiveness and health services research, and clinical trials[3, 5]. The Canadian Institutes of Health Research (CIHR), among other research organizations, have outlined and endorsed the use of administrative health databases for outcomes research as one strategy for enhancing patient-oriented research[6] and improving health care efficiency and delivery. However, as with any new research tool, the limitations, biases, and methods associated with research using routine health data have raised increasing concerns[4]. Adequate and clear reporting of research methods and results is needed to enable the research consumer to judge studies′ strengths and limitations.

At present, researchers who conduct observational research are encouraged to use the **STR**engthening the **R**eporting of **OB**servational studies in **E**pidemiology **(STROBE)** statement as guidance when reporting their research[7]. Transparent reporting facilitates decision making by all readers and reproducibility of methods by interested researchers.[8] However, the rapid evolution and availability of routinely collected data for research has brought to light specific issues not addressed by STROBE. This gap was acknowledged by a large group of scientists (including five members of the STROBE Steering Committee) at a meeting following the 2012 Primary Care Database Symposium (27 January 2012 in London, UK). The group identified the need to expand the STROBE statement to encompass studies based on routinely collected health data, most of which are observational (non-randomized) in design. Since stakeholders in research relying on routine health data are diverse (including researchers, clinicians, health policymakers, and representatives of the pharmaceutical industry), meeting attendees recommended that a wide range of stakeholders participate in expanding the reporting guidelines to ensure adequate representation of interests and views.

Since not all stakeholders can attend a face-to-face meeting to create reporting guidelines, a Delphi exercise is often used to obtain information to inform those who write the guidelines.[9] Such an exercise can be conducted using web and social media technologies to allow for the creation of a document that incorporates a large and diverse group of stakeholders. The purpose of the project reported here was to determine the interests and priorities of stakeholders in research conducted using routine health data, in order to guide the development of the **RE**porting of studies **C**onducted using **O**bservational **R**outinely-collected health **D**ata (**RECORD**) statement, an extension of the STROBE statement. We sought to identify issues important to stakeholders in order to create the most representative set of reporting guidelines possible.

## Methods

**Fig 1:**
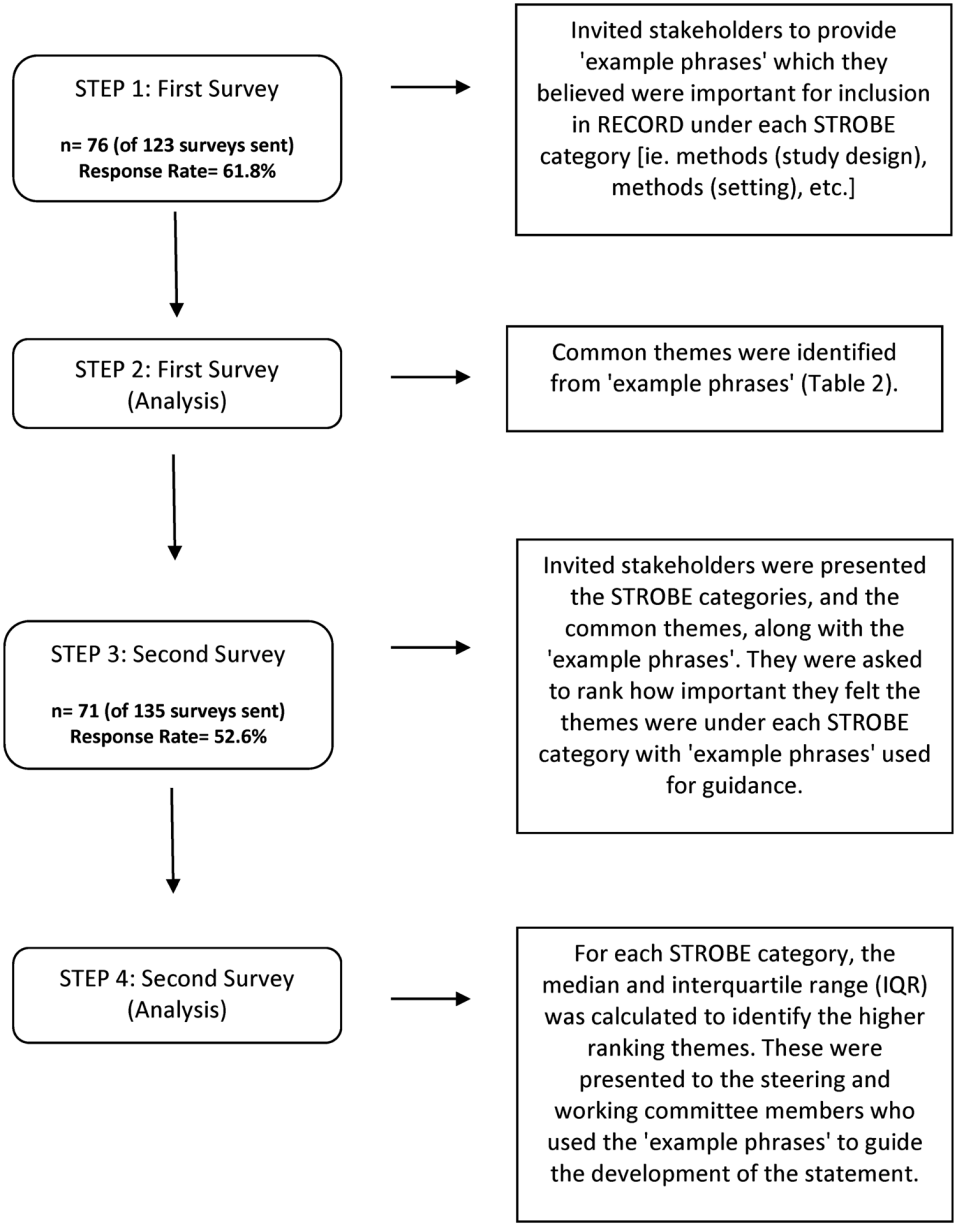
Flow diagram of steps used to elicit stakeholder priorities for RECORD.

We used a three-stage process to develop an extension of the STROBE guidelines specific to observational studies conducted using routinely collected health data, *i*.*e*., the RECORD statement (record-statement.org). The first stage of the process was a modified Delphi exercise to elicit the priorities of a large, diverse group of stakeholders through two surveys (Fig 1). The second stage consisted of a face-to-face meeting of the RECORD working committee members. It reviewed the survey results and processed stakeholder recommendations in order to create checklist items and explanatory text. The third stage of the process consisted of review of the draft checklist and explanatory document posted in an online message board on the RECORD website (record-statement.org) by a wide group of research stakeholders.

### Participants in the surveys

Eligible survey participants were stakeholders in the broad context of use of routinely collected health data for research, including both researchers and users of research results. Stakeholders included, but were not limited to, clinicians, clinical and academic researchers, biomedical journal editors, policymakers, and pharmaceutical industry representatives.

Multiple methods were used for recruitment. Members of the RECORD Steering Committee each identified 5–10 experts in the field, who were contacted directly. Additional participants were recruited through snowball sampling[10], in which the initially invited participants were asked to identify others. In addition, stakeholders were recruited through appropriate electronic mailing lists (listservs) including the Cochrane Collaboration, AcademyHealth, and the Agency for Healthcare Research and Quality. Interest was also garnered through editorials in the *Journal of Clinical Epidemiology*[11] and *Clinical Epidemiology*[12]. The editorials outlined the need for expanded reporting guidelines, requested stakeholder involvement, and provided contact information for the study. Stakeholders′ interest in research based on routinely collected health data was ascertained in order to ensure the relevance of their input. All stakeholders who expressed an interest were included in the surveys. See S1 Appendix for a complete list of stakeholders who participated in various stages of the surveys and provided message board feedback.

### Surveys

A two-stage modified Delphi process was used to generate items for inclusion in the RECORD guidelines and to rank responses. The first stage was used to generate an extensive list of potential items, and the subsequent stage focused on reducing and prioritizing these items through a consensus process of rating each item in terms of its importance.

**Fig 2:**
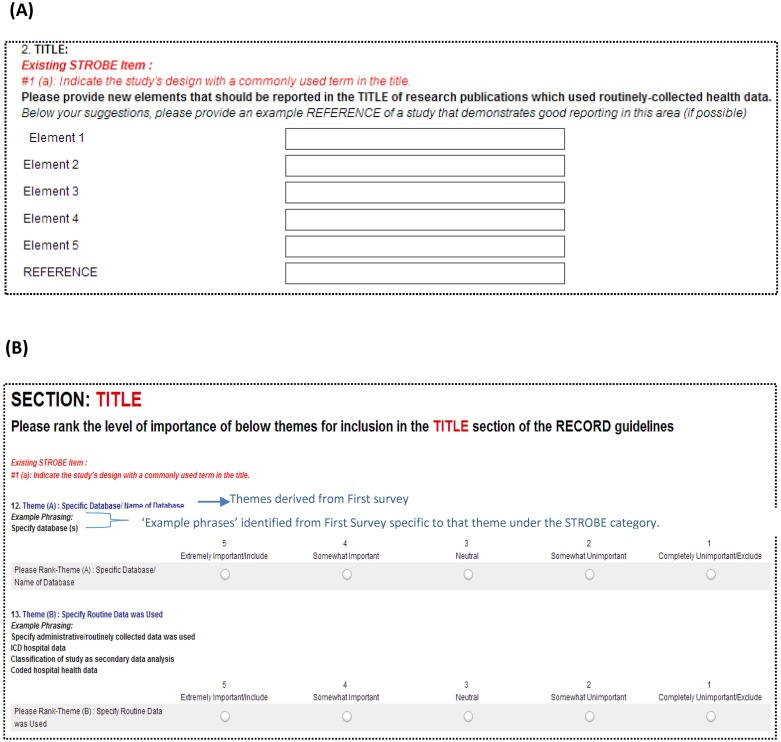
Examples of layout from (A) first survey (free-text responses) and (B) second survey (Likert-scale quantitative ranking).

In the first survey round, participants were asked to identify specific themes that should be included in the RECORD reporting guidelines (see example question Fig 2A). This question sought to generate overall themes that are important to consider in the reporting of research based on routinely collected health data, and allowed participants to propose broad groupings of items. Participants also were presented with existing the STROBE guideline categories[7] [*e*.*g*., title and abstract, introduction (background/rationale), introduction (objectives), methods (study design), methods (setting), etc.] and were asked to list additional items needed to report research based on routinely collected health data and thus important for inclusion in the RECORD statement. Open-ended free text responses were collected to allow for full elaboration of meaning and rationale.

In the second survey round, respondents received a list of themes emerging from the first round (see example in Fig 2B). In order to provide clear examples to participants of the items relating to each theme, the second survey presented themes with example phrases of individual components. For both the overarching themes and items within existing STROBE categories, rating was performed using a 5-point Likert scale (1-strongly disagree to 5-strongly agree).

Links to the online surveys were emailed to participants with a specified deadline. Reminder emails were sent one week prior to the deadline, and a two-week extension was provided for both stages to ensure maximum participation. Both surveys were conducted using SurveyMonkey (surveymonkey.com, Palo Alto, CA, U.S.A.) and ethics approval was granted by the Children′s Hospital of Eastern Ontario (CHEO) Research Ethics Board (#13/45X).

### Data Analysis of Surveys

Suggestions and comments procured from the first stage were imported into Qualitative Data Analysis Software (QDA) for analysis. Comments provided by respondents under each section of the STROBE checklist were coded using a process of qualitative description.[13] In this low-inference approach to coding qualitative data, the focus was a descriptive account of the text, as opposed to generation of theory. This was in keeping with our objectives for the Delphi process, *i*.*e*., to group elements into broad themes or constructs and to rank them, rather than to develop a theory concerning attitudes towards reporting of studies using routinely collected health data.

Initial coding for both overarching themes and category-specific items was undertaken by one investigator (SN). The initial list of themes and items then was refined and reduced through discussion with the steering committee. The themes generated within each STROBE category were then compiled across categories to provide an overall list of themes to be considered in the reporting guideline extension.

During the second (quantitative) stage of the Delphi process, we determined the mean, median, and interquartile range (IQR) for individual themes under each STROBE category and a rank order for each category. This step provided a prioritized list of themes on which to base the RECORD guidelines.

### Working Committee Meeting

The working committee (which included members of the steering committee, journal editors, researchers, and other stakeholders) met in Lausanne, Switzerland from the 22^nd^ to 24^th^ of October, 2013. Working committee members consisted of three groups: 1) internationally recognized scientists who use routinely collected health data for research, 2) members of the STROBE working committee and/or EQUATOR Network who are experts in reporting guideline development, and 3) editors from journals which frequently review and publish observational research using routinely collected health data (*BMJ*, *CMAJ*, *Health Services Research*, *PLoS Medicine*, with input *in absentia* from editors of *Clinical Epidemiology* and *Journal of Clinical Epidemiology*). A list of working committee members is presented in S2 Appendix. The list of themes produced by the stakeholder surveys was presented in ranked order by observational research theme (as categorized in STROBE) to all members. The committee was divided into working groups by theme and asked to review stakeholder comments and quantitative rankings from the surveys. The committee created draft statements and themes for inclusion in the RECORD checklist and explanatory document. The full working committee then reviewed these draft statements and voted on agreement. Statements were discussed and revised until >80% agreement was achieved. Live polling was conducted using Poll Everywhere (polleverywhere.com, San Francisco, CA).

### Post-Meeting Evaluation of Draft Statements

The draft RECORD statement written by the working committee and explanatory documentation were made available for stakeholder review from the 8^th^ of September to 14^th^ of November, 2014. The draft checklist items with explanatory text were posted on a password-protected message board on the website record-statement.org. Checklist items were grouped by STROBE categories. Stakeholders were assigned a username and password and encouraged to engage in discussion. Those who did not provide written comments on a statement were asked to rank the statement on a 10-point scale.

## Results

Fig 1 provides a flow diagram of steps used to assess stakeholder priorities for RECORD. For the first survey, 98 stakeholders, nine steering committee members, and 16 working committee members were invited to complete the survey during April and May 2013. Of the 123 potential participants, 76 responded (response rate of 61.8%), and, of these, 68 responded to the optional demographic questions. For the second survey, 106 stakeholders, nine steering committee members, and 20 working committee members were invited to complete the survey between July and September 2013. Of the 135 potential participants, 71 responded to the second survey (response rate of 52.6%), and, of these, 56 responded to the optional demographic questions. Participant demographics are shown in Table 1.

**Table 1:** Characteristics of respondents.

	First Survey (n = 68)^a^	Second Survey (n = 56)^b^
	n (%)	n (%)
**Sex**		
Male	37 (54.5%)	28 (50.0%)
Female	31 (45.6%)	28 (50.0%)
**Age (years)**		
18–34	8 (11.8%)	2 (3.6%)
35–49	39 (57.4%)	36 (64.3%)
50–64	20 (29.4%)	16 (28.6%)
65+	1 (1.5%)	2 (3.6%)
**Country of Residence**		
United Kingdom	34 (50.0%)	24 (42.9%)
United States of America	7 (10.3%)	5 (8.9%)
Canada	7 (10.3%)	7 (12.5%)
France	4 (5.9%)	5 (8.9%)
Germany	3 (4.4%)	3 (5.4%)
Italy	3 (4.4%)	2 (3.6%)
Australia	2 (2.9%)	3 (5.4%)
Switzerland	2 (2.9%)	2 (3.6%)
Sweden	1 (1.5%)	0
Denmark	1 (1.5%)	0
The Netherlands	1 (1.5%)	2 (3.6%)
New Zealand	1 (1.5%)	0
Brazil	1 (1.5%)	1 (1.8%)
Finland	1 (1.5%)	0
Uganda	0	1 (1.8%)
Romania	0	1 (1.8%)
**Primary profession**		
Researcher (MD)	26 (32.8%)	22 (39.3%)
Researcher (PhD, MSc., BSc., etc.)	37 (54.4%)	31 (55.4%)
Journal editor, journal administrative staff	2 (2.9%)	3 (5.4%)
Public (non-medical, non-researcher) stakeholder	1 (1.5%)	0
Policymaker	1 (1.5%)	0
Pharmaceutical industry representative	1 (1.5%)	0
**Type of routinely collected health data used for research** ^**c**^		
N/A (not a researcher)	3 (4.4%)	3 (5.4%)
Health administrative data (government)	35 (51.5%)	32 (57.1%)
Health administrative data (insurance provider)	14 (20.6%)	13 (23.2%)
Health administrative data (other)	17 (25.0%)	9 (16.1%)
Cancer registry	18 (26.5%)	12 (21.4%)
Disease registry	27 (39.7%)	26 (46.4%)
Electronic health/medical records	44 (64.7%)	30 (53.6%)
Government statistical databases (excluding health)	17 (25.0%)	16 (28.6%)
Primary care databases (*e*.*g*. GPRD, CPRD, IPCI)	33 (48.5%)	27 (48.2%)

^a^ Sixty-eight out of 76 respondents provided information on demographics (an optional question).

^b^ Fifty-six out of 71 respondents provided information on demographics (an optional question).

^c^ Respondents were able to select more than one option.

### First Stage Survey (Qualitative Analysis and Development of Themes)

Open-text responses for both the overarching themes and STROBE-specific categories indicated a focus on methodological issues. Comments reflected a need for information not only on the specific contents of the database and the proposed operational definitions, but also other contextual information such as whether the data were drawn from a publicly funded healthcare system or one based on private medical insurance.

The first stage open-text survey generated 311 responses relating to overarching themes to be included in the RECORD statement. These were collated into 131 individual codes. From these codes, a total of 10 overarching themes were derived. A list of the overall themes, together with examples of items coded within the themes, is provided in Table 2.

**Table 2:** Overall themes suggested by respondents in the first survey and mean ratings from the second survey.

Overall Theme	Frequency^a^	Example Phrasing^b^	Mean Rating^c^ ^, ^ ^d^
Characteristics of the data itself- quality, data source/setting, type of database, data collection process	66	Data completeness; Purpose of the recorded data; Data collection process; Description of datasets	**4.63**
Validity of diagnostic codes for outcomes of exposures	59	Validity; Validity (codes); Validity (procedures); Validity of outcomes; Data quality (data checks)	**4.56**
General methods (methods, analysis, confounding)	35	Confounding; Rationale for research; Bias; Bias (time); Confounders (measurement of)	4.36
Disease/exposure identification algorithms (excluding validation)	32	Disease identification algorithm; Exposure (definition of); Algorithm specification; Case definition; Code (selection)	**4.78**
Linkage	31	Data linkage; Data linkage (procedures); Data linkage (success rates); Data linkage (quality of); Matching algorithms	**4.62**
Characteristics of the population included in the data- including geographic region, population included, sampling	30	Coverage of dataset; Population (definition of); Population covered; Dataset characteristics described (or reference provided); Sampling	**4.76**
List of Codes	23	Code list; Codes (type of); Setting and sampling; Diagnosis codes; Codes (definition of)	4.18
Missing Data- How was missing data handled? Why? Proportion?	15	Missing data; Missing data (approaches to deal with); Administrative data methods used (imputation, etc.); Bias (missing data); Missing data (proportion of)	4.43
Ethical/legal/access/availability	11	Availability of databases being used; Access to data codebooks; Data security issues; Governance issues; Legal access to the database	3.76
Uncategorizable	8	Temporal relationships; Variable generation; Abstract; Code (impact of)	3.50
Identify as routine data study	1	Description of study as routine data study	4.21

^a^ Number of respondents providing a phrase in this category in the first-stage survey.

^b^ List of 'example phrases' from the first stage survey with the highest frequencies.

^c^ Mean score from the second stage survey is provided. 5-Strongly agree for importance for inclusion; 1- Strongly disagree for importance for inclusion.

^d^ Top five themes from the second-stage survey with the highest means are bolded.

Within each original STROBE category, the total number of responses and codes varied, ranging from 15 responses generating 10 unique codes for the STROBE category ‘*Results-Main Results’*, to 149 responses generating 86 distinct codes for the category ‘*Methods-Setting’*. Compiling the themes generated within each STROBE category provided a total of 13 themes (Table 3). These themes broadly reflected the overarching themes, with the majority of themes being associated with relevant STROBE categories.

**Table 3:** Mean rating of themes by manuscript section, as defined by the STROBE reporting checklist.

Theme	Title	Abstract	Introduction	Methods (Study design)	Methods(Setting)	Methods (Participants)	Methods (Variables)	Methods (Data sources)	Methods (Bias)	Methods (Study Size)	Methods (Quantitative Variables)	Methods (Statistical Methods)	Results (Participants)	Results (Descriptive)	Results (Outcome Data)	Results (Main Results)	Results (Other Analyses)	Discussion (Key Results)	Discussion (Limitations)	Discussion (Interpretation)	Discussion (Generalizability)	Other
Specify database (name)	**3.19**	-	3.33	**4.28**	-	-	-	**4.60**	-	-	-	-	-	-	-	-	-	-	-	-	-	-
Specify that routine data were used	**3.66**	**4.42**	-	**4.57**	-	-	-	-	-	-	-	-	-	-	-	-	-	-	-	-	-	-
Type of routine data used	**3.53**	**4.52**	**3.46**	**4.32**	**4.16**	-	-	-	-	-	-	-	-	-	-	-	-	-	-	-	-	-
Geographic area, setting, population	**3.58**	**4.43**	**3.56**	3.89	**4.77**	**4.57**	2.93	3.76	3.53	**4.02**	-	-	**4.40**	**4.53**	**3.93**	**3.32**	-	-	4.28	**3.81**	**4.49**	-
Linkage	2.95	**3.85**	**-**	4.13	**3.51**	3.48	3.42	**4.25**	**3.92**	-	-	**3.78**	**4.25**	**4.05**	**3.39**	-	**3.88**	-	4.26	**3.91**	-	-
Validation	3.00	3.45	3.09	4.05	3.18	**3.59**	**4.30**	**4.00**	**4.00**	-	-	**3.88**	**3.44**	**3.60**	**3.86**	-	**3.81**	**3.98**	**4.46**	**3.91**	**3.53**	2.84
Coding/classification used	2.48	3.32		**4.21**	3.33	**3.98**	**4.58**	**3.92**	**3.80**	-	**4.05**	-	3.41	-	**3.57**	-	-	**-**	4.28	3.42	-	**3.49**
Rationale for registry/routine data collection approach	-	-	**4.09**	**3.55**	**-**	**-**	-	-	-	-	-	-	-	-	-	-	-	**4.25**	**4.35**	**3.71**	**3.72**	**2.98**
General methods (methods, analysis, confounding)	**3.82**	**4.28**	**-**	**4.60**	**3.34**	**3.78**	**3.78**	3.56	**4.27**	**4.05**	**4.24**	**4.44**	**3.88**	**3.53**	**4.26**	**4.44**	**4.21**	**4.32**	-	**4.14**	**4.04**	-
Specify objectives	-	-	**4.50**	3.71	**-**	-	-	-	-	-	**4.05**	**3.98**	-	-	-	**3.47**	**3.81**	**4.11**	-	-	-	-
Methods used to get data into analyzable format (*e*.*g*., data cleaning)	-	-	**4.00**	3.62	2.93	3.33	**3.53**	3.59	3.48	-	-	3.37	-	-	3.37	-	-	-	**4.32**	-	-	**3.60**
Ethics, legality, access, availability	-	-	-	3.71	2.95	2.67	-	3.23	-	-	-	-	-	-	-	-	-	-	3.07	-	-	**3.88**
Characteristics of the data-quality, data source/setting, data collection	-	3.42	-	3.92	**3.69**	**3.60**	**3.95**	**4.25**	**4.17**	**3.68**	**4.10**	**3.39**	**3.75**	**3.51**	3.28	**3.18**	-	**3.77**	**4.47**	3.65	**4.12**	**3.39**

STROBE sections with "-" for a theme indicate that no "example phrases" related to that theme were provided under that STROBE category in the first survey. Top five themes from the second survey with the highest means are bolded.

Respondents ranked themes on a 5-point Likert scale to determine whether they agreed with inclusion in the final checklist (5-Strongly agree for importance for inclusion; 1- Strongly disagree for importance for inclusion.)

### Second Stage Survey (Quantitative Analysis of Themes)

Ratings from the second stage survey indicated that priorities for RECORD checklist items centered on reporting of *methodological aspects*, as opposed to reporting of *results*. The highest rated overall themes for inclusion in the RECORD reporting guidelines were: (i) Disease/exposure identification algorithms (mean 4.78); (ii) Characteristics of the population included in databases (mean 4.76); (iii) Characteristics of the data (mean 4.63); (iv) Linkage (mean 4.62); and (v) Validity of diagnostic codes (mean 4.56) (see Table 2).

Within existing STROBE categories the importance assigned to each of the compiled themes varied, indicated by the mean rating. For example, describing the ‘Characteristics of data quality, data source/setting, data collection’ was rated more highly in relation to the STROBE categories of *Methods (Bias)* (mean 4.17) and *Discussion (Limitations)* (mean 4.47), than it was for *Methods (Setting)* (mean 3.69) or *Results (Outcome Data)* (mean 3.28). Table 4 presents summary data on the top-rated themes within each existing STROBE category. The raw data for both surveys is also available on the RECORD website (http://record-statement.org/surveyrawdata or Data available from the Dryad Digital Repository: http://dx.doi.org/10.5061/dryad.7d65n).

**Table 4:** Most highly rated themes by manuscript section.

Manuscript Section	Highest Ranking Items	Sample of ‘Example Phrases’ under Theme		Ranking of Theme	
			Mean	Median	IQR
**Title**	General Methods	Indicate key findings	3.82	4	2
	Specify that routine data were used	Specify administrative/ routinely collected data was used	3.66	4	2
**Abstract**	Type of routine data used	Specify/describe data source(s) used	4.52	5	1
	Geographic area, population setting	Describe study setting; Describe time period covered	4.43	5	1
**Introduction**	Specify objectives	Specify objectives	4.50	5	1
	Rationale for registry/routine data collection approach	Rationale for routine data collection approach; Justify selection of specific database(s)	4.09	4	1.25
**Methods**					
Study design	General methods	Indicate whether retrospective or prospective; Describe how missing data was dealt with; Specific if self controlled case series (SCCS) or other methods were used	4.60	5	0
	Specify that routine data were used	Make reference to routinely collected data	4.57	5	0
Setting	Geographic area, population setting	Time period for which data was retrieved and time period that database covers; Describe population covered by data source	4.77	5	0
	Type of routine data used	Purpose of the database (*e*.*g*. claims, etc.).	4.16	5	1
Participants	Geographic area, population setting	Describe how the cohort was constructed; Flow diagram of how population was narrowed down	4.57	5	1
	Coding used	Algorithm do identify individuals; Code list for diagnoses	3.98	4	2
Variables	Coding used	Share code list in supplement (when feasible); Describe diagnostic algorithms	4.58	5	1
	Validation	Validation of code list; Describe validity of the variables	4.30	5	1
Data Sources/ Measurement	Specify database	Define data source	4.60	5	0
	Linkage	Describe linkage methods and their accuracy and quality	4.25	5	1
	Characteristics of the data	Data collection methods; Database creation and maintenance			
Bias	General methods	Describe methods to address confounding due to unmeasured variables; Discuss immortal person time bias; Discuss bias due to missing data	4.27	5	1
	Characteristics of the data	Bias due to the purpose of original data collection; Bias in source data; Changes in codes over time	4.17	4.5	1
Study Size	General Methods	Specifying the power based on size of the database; Being aware that clinical relevance is more important than sample size and to interpret P values with caution	4.05	4	1.25
	Geographic area, population setting	Providing details on whether the complete population was used or just sample (convenience sample); Describing any reduction in sample size due to matching	4.02	4	1
Quantitative Variables	General methods	Identifying whether case-based or person-based analyses were done; Explaining how time-varying variables were handled; Describing management of missing data	4.24	5	1
	Characteristics of the data	Completeness of the data; Were cut-offs pre-specified in the database or later by the researchers?	4.10	4	1
Statistical Methods	General methods	Describe how clustering was dealt with; Assessment of multi-collinearity	4.44	5	1
	Specify objectives	Indicate that there was a pre-analysis statistical plan	3.98	4	1.5
**Results**					
Participants	Geographic area, population setting	Flow diagram to illustrate study numbers of each database used at each stage; Indicate number of missing due to missing code	4.40	5	1
	Linkage	Number of cases linked, and number of cases excluded due to non-linkage; Comment on differences between linked and non-linked individuals; Comment on linkage quality	4.25	5	1
Descriptive	Geographic area, population setting	Describe censoring (as a result of leaving the healthcare system)	4.53	5	1
	Linkage	Similar ‘example phrases’ from results (Participants)	4.05	4	0
Outcome Data	General Methods	SCCS, report number of outcome events in exposed patients	4.26	5	1
	Geographic area, population setting	Number in each outcome group	3.93	4	2
Main Results	General methods	Provide appropriate P values for sample sizes (e.g. P <0.01 may be more appropriate); Translate effect size back into units which readers will understand	4.44	5	1
Other Analyses	General methods	Report assessment of collinearity for categorical variables; Report assessment of correlation for categorical co-variables; Report sensitivity analyses (if there is reason to suspect differential misclassification)	4.21	4	1
	Linkage	Provide any estimates of bias from linkage	3.88	4	2
**Discussion**					
Key Results	General Methods Rationale for	Comparisons with other possible sources of data	4.32	5	1
	Routine Data Collection Approach	Specifying importance of kind of database used	4.25	4	1
Limitations	Characteristics of the Data	Limitations of the data source (quality of the data source, bias in original data source); Database coverage of the population; Completeness of the database; Impact of changing practice in data collection or coding on results	4.47	5	1
	Validation	Degree of validity of the data; Accuracy of the coding used	4.46	5	1
Interpretation	General Methods	Coherence, comparability, and consistency to other studies; Validity of conclusions drawn (from using poor quality data)	4.14	5	1
	Linkage	Potential bias a result of differential linkage in subgroups	3.91	4	2
Generalizability	Geographic area, population setting	Describe how participating practices/health care providers are representative of those in that country; Completeness of coverage (which patients/providers are missed)	4.49	5	3
	Characteristics of the Data	Generalizability with reference to other databases	4.12	4	1
**Other**	Ethics/Legality/Access/Availability	Address funders of the dataset; Address any data sharing issues (i.e. their availability); Address roles of the data custodians; Justify lack of open availability (if not open to other researchers); Discuss privacy protection	3.88	4	2
	General Methods	Include technical appendix	3.60	4	1

Respondents ranked themes on a 5-point Likert scale to determine whether they agreed with inclusion in the final checklist (5-Strongly agree for importance for inclusion; 1- Strongly disagree for importance for inclusion.)

### Working Committee Meeting and Post-Meeting Feedback

During the working committee meeting, its members processed and prioritized stakeholder comments to create the draft RECORD checklist. Consensus of >80% was achieved for each checklist item. The resulting checklist will be made available in the main RECORD publication and on the RECORD website (record-statement.org) along with explanations and examples for each item. Working committee meeting minutes are available upon request.

Following the working committee meeting, draft checklist items and explanatory text were posted on a message board on record-statement.org for review by stakeholders. During the review period, 311 users accessed the website. Of these, 66.5% were new users. The most common countries of origin of website users were United Kingdom (20.6%), Brazil (14.5%), Canada (14.5%), Italy (11.9%), and the United States (9.8%) (Google Analytics for website record-statement.org). Ratings by message board participants are provided in Table 5.

**Table 5:** Summary of post-meeting message board activity for draft statements.

Statement Category	Mean Ranking	Number of Comments	Number of Views
**1. TITLE/ABSTRACT**	7.5	10	77
**2. INTRODUCTION (OBJECTIVES)**	5.8	13	103
**3. METHODS (PARTICIPANTS)**	7.5	14	85
**4. METHODS (PARTICIPANTS, LINKAGE)**	8.3	5	84
**5. METHODS (VARIABLES OR DATA SOURCES)**	9.2	8	131
**6. METHODS (STATISTICAL ANALYSIS)**	7.5	18	125
**7. RESULTS (PARTICIPANTS)**	10.0	7	85
**8. DISCUSSION (LIMITATIONS)**	6.7	12	87
**9. OTHER INFORMATION**	8.3	6	90

Rankings were assigned (out of 10) by participants who did not respond with a written comment.

## Discussion

The quality of reporting of research based on routinely collected health data has been suboptimal [14, 15], potentially resulting in misinterpretation or misapplication of research findings. We conducted two inclusive, comprehensive surveys of stakeholders to determine the topics of highest priority for inclusion in the RECORD guidelines checklist for studies using routinely collected health data. The results of these surveys have been used to ensure that RECORD guidelines adequately reflect the priorities of individuals who make scientific, policy, and clinical decisions based on these data. Our findings point to unique aspects of studies conducted with routinely collected health data and confirm the need for clarity regarding reporting of methodological issues.

Reporting guidelines most often take the form of a checklist, flow diagram, or explicit text designed to assist authors in reporting a specific type of research. This may allow for transparency and reproducibility of research methods and findings. They may also improve the completeness of reporting research.[16] Reporting guidelines have been demonstrated to improve reporting of studies when they are adopted by the research community and relevant journals,[17–20] although their effectiveness varies[20, 21] and may depend on implementation by journals rather than simple endorsement.[12] Creation of guidelines typically involves consensus to determine a minimum set of criteria for reporting research [22, 23]. Concomitantly, adherence to and implementation of guidelines may aid in the conduct of systematic reviews and meta-analyses.

In response to the increasing number of reporting guidelines available or under development, the Enhancing the QUAlity and Transparency of health Research (EQUATOR) Network has published guidance on how to develop a reporting guideline[9]. The document recommended a Delphi survey as part of the development process to allow inclusion of participants unable to attend face-to-face meetings. RECORD undertook expansive surveys of stakeholders and social media to solicit the opinions of a large, geographically diverse group of stakeholders from multiple disciplines. This work has allowed us in order to prioritize the draft items of a reporting guideline checklist to better reflect the needs of these stakeholders.

The qualitative data generated by participants in our Delphi survey were illuminating in terms both of the specific nature of comments received, and their implications for topics to be emphasized in the RECORD guidelines. In particular, the survey generated many comments regarding broad themes for reporting of methodology, but far fewer comments regarding reporting of results or discussion. This emphasis on the methodological aspects of research may reflect the fact that RECORD was considered as an extension of the original STROBE statement. Respondents may have perceived that the strength of RECORD would lie in addressing specific methodological concerns regarding research based on routinely collected health data that were not addressed within the more general STROBE statement. Prioritization of data linkage methods and code validation reflects the activity of the research community in these fields, including work performed by members of the RECORD group[24, 25]. Other themes represent new concerns not previously reported in the literature, such as the need to report on characteristics of the database itself, the underlying population from which the database was derived, and issues of governance and availability of the data to other researchers. Finally, some themes prioritized by respondents represented general research methods not specific to studies based on routinely collected health data (*e*.*g*., missing data, potential confounding, and data security issues), but which are particularly salient in this form of research. While the RECORD guidelines addresses as many concerns as possible, it will remain specific to studies conducted using routinely collected health data. The two surveys described above allowed the RECORD working committee to prioritize checklist items for inclusion in the RECORD statement and to guide the placement of the items within the publication′s structure.

### Limitations

A limitation of the modified Delphi approach is that findings are specific to the group of individuals surveyed and may not necessarily represent popular opinion among the broad range of stakeholders. In order to strengthen the validity of our results, we identified potential survey participants using active and passive selection. The actively selected group was approached based on expert recommendation, to ensure breadth of experience and interest in the methodological and substantive content of research publications. The passively selected group approached us following a wide-ranging awareness campaign. One limitation that may be levelled at the stakeholder group is the apparent dominance of academic scholars. However, this belies the multiple roles occupied by participants, with many respondents also holding editorships within journals serving the target population of end users of health administrative data, as well as individuals who serve policy makers in consultative capacities. As such we believe that the stakeholder composition is robust with respect to both academic rigor, but also in terms of encouraging quality decision-making within a learning healthcare environment. In particular, a strength of our approach was the commitment of panelists in completing the Delphi process. A limitation was that the stakeholder group originated predominantly from North America, Europe, and Australia, although participation was possible without regard to geographic region. English-language advertisements and editorials requesting participation may explain this pattern of input. As well, the majority of medical research using routine health data originates from these regions. We thus believe that our stakeholder group is representative of the broad community of researchers using such data. It is noteworthy that the most frequent visitors to the record-statement.org website were from both English and non-English speaking countries.

Use of a password-protected message board to elicit post-meeting feedback may have also limited the generalizability of this feedback. However, we aimed to receive comments on the draft statements from the same stakeholder group who provided feedback prior to statement creation. Approximately 10% of persons viewing the draft statements on the message board provided written comments, while the remainder gave numerical ratings. This level of contribution follows the “1% rule” of internet culture, *i*.*e*., that 1% of internet users create content, 9% of users modify or comment on that content, and 90% of users consume or observe internet activity.[26] Despite this, the contributions to the message board led to valuable pre-publication discussion and revision.

Another potential limitation involved use of a traditional working committee meeting to draft the checklist and explanatory document. While we attempted to select the working committee from a wide range of stakeholders, particularly those with the greatest enthusiasm during the survey stages, only 17 committee members could participate in the face-to-face meeting. However, survey responses reflected the interests and input of the larger stakeholder group. Future reporting guideline initiatives should consider use of more advanced forms of social media and internet-based conferencing to ensure involvement of a broader group of stakeholders in creating the checklist.

### Next Steps—the RECORD Statement

In summary, we elicited input from a broad range of stakeholders to specify priorities for a definitive reporting guideline checklist for research conducted using routinely collected health data. The RECORD statement incorporated the suggestions provided by stakeholders to create a reporting checklist specific to observational research using routine health data. The draft checklist and explanatory document were made available to the stakeholder group for comment and revision via online message board.

While participants reflected a range of academic and non-academic roles across a range of health-related interests, post-publication activities will seek to further engage stakeholders from a range of perspectives, including journal editors, policy decision-makers, and those involved in the development of health administrative datasets. Following peer review and publication, the final RECORD checklist will be available for comment on the RECORD website. We will also seek the endorsement of the guidelines by appropriate journals, utilizing the stakeholder membership to facilitate this process, while undertaking an active dissemination process presenting the guidelines at appropriate academic and non-academic meetings. This will include translation of the guidelines into non-English language versions to enhance uptake internationally. Furthermore, we will evaluate the impact of the RECORD guidelines through a planned systematic review that will allow the group to compare adherence within articles published by journals that do and do not endorse the guidelines. Using these diverse methods, we anticipate that RECORD will improve the clarity of reporting of research conducted using routinely collected health data.

## Supporting Information

S1 Appendix List of stakeholders who participated in the two surveys. (XLSX)

S2 Appendix List of working committee members. (XLSX)
